# Intraoperative radiation therapy in the treatment of early-stage breast cancer utilizing xoft axxent electronic brachytherapy

**DOI:** 10.1186/1477-7819-7-24

**Published:** 2009-03-02

**Authors:** Adam Dickler, Olga Ivanov, Darius Francescatti

**Affiliations:** 1 Department of Radiation Oncology, Little Company of Mary Hospital, Evergreen Park, IL 60805, USA; 2 Department of General Surgery, Little Company of Mary Hospital, Evergreen Park, IL 60805, USA; 3 Department of General Surgery, Rush University Medical Center, Chicago, IL 60612, USA

## Abstract

**Background:**

In an effort to overcome the barriers to BCT, alternative methods of delivering radiation therapy have been explored. APBI allows the radiation treatment to be accomplished in one week or less. XB is a form of balloon-based APBI that uses an electronic source generated by a mobile controller unit. Investigators have also explored IORT treatment that delivers a single fraction of radiation in the operating suite at the time of surgery.

**Methods:**

We report on the first patient treated with XB to deliver IORT.

**Results:**

IORT treatment utilizing XB is feasible and can be accomplished with a total procedure time of approximately 2 hours.

**Conclusion:**

Further research on XB and other methods of IORT is needed to establish clinical efficacy and safety for patients with early-stage breast cancer.

## Background

Several large randomized trials with extended follow-up have shown that breast conserving therapy (BCT) offers equivalent overall survival to modified radical mastectomy in patients with early stage breast cancer [1,2]. Studies have also shown that in certain parts of the United States, as few as 10% of eligible patients receive BCT [3,4]. Instead, some women are treated with mastectomy and others receive lumpectomy alone. This is especially common for women who live at an increased distance from radiation centers [5,6]. The logistical difficulties that accompany a 6–7 week course of whole breast external beam radiation (EBRT) are believed to be the main deterrent to patients receiving radiation therapy. Although radiation therapy is burdensome, it is an important component of BCT that cannot be safely omitted. EBRT has been shown to both decrease the risk of local recurrence and also to improve overall survival compared to surgery alone [7].

In an effort to overcome the barriers to BCT, alternative methods of delivering radiation therapy have been explored. In contrast to standard EBRT, which treats the whole breast, accelerated partial breast irradiation (APBI) delivers radiation to the lumpectomy bed plus a 1–2 cm margin only. By decreasing the volume of treatment and increasing the daily fraction size of the radiation, treatment can be accomplished in one week rather than the standard 6–7 weeks.

The method of APBI with the longest published experience is multi-catheter interstitial brachytherapy. In this technique, several rows of catheter needles are placed around the lumpectomy bed and loaded with radiation sources. Multi-catheter interstitial brachytherapy has been associated with local recurrence rates of less than 5% at over 5-years follow-up and favorable cosmesis [8,9]. Unfortunately, this technique is difficult and requires significant expertise. As a result, multi-catheter interstitial breast brachytherapy is performed at relatively few centers in the U.S.

Balloon-based APBI methods were developed to simplify the brachytherapy procedure. The MammoSite™ (MS) brachytherapy applicator was the first balloon catheter to be developed for APBI. The catheter contains an inflation channel and a channel for the passage of an Iridium-192 (Ir-192) radiation source. It can be inserted through a single incision at the time of surgery or post-lumpectomy using ultrasound guidance. Initial results regarding cosmesis and local control using the MS catheter have been comparable to multi-catheter interstitial brachytherapy [10,11].

Additional methods of balloon-based APBI are also being explored. A modified form of balloon-based brachytherapy called Xoft Axxent Electronic Brachytherapy™ (XB) received FDA clearance for the treatment of breast cancer in January, 2006. This device uses a mobile controller, which generates kilovoltage (kV) x-rays. This approach to APBI requires minimal shielding and thus has the potential to increase the number of settings in which radiation treatments can be offered. In addition, XB is not limited by rigorous radiation source regulations associated with other methods of APBI, which utilize radioisotope sources. The early results of a clinical trial to evaluate the performance and safety of XB in the outpatient treatment of early-stage breast cancer patients were recently presented at the American Society of Clinical Oncology (ASCO) Breast Cancer Symposium. Treatment with XB was found to be feasible and associated with minimal acute side effects [12].

Investigators have also explored delivering APBI in the operating room immediately after lumpectomy. Intraoperative radiation therapy (IORT) allows the patient to receive all her radiation in a single fraction before she awakens from surgery. Additional potential advantages include delivering the radiation before tumor cells have a chance to proliferate, performing the radiation under direct visualization at the time of surgery, and decreasing healthcare costs. Published results using IORT both as a tumor bed boost in conjunction with EBRT and as a primary treatment in APBI have shown favorable outcomes [13-15].

To date, XB has been utilized only in an outpatient setting to deliver APBI in 10 fractions over 5 days. We report on the first patient treated using XB to deliver IORT as part of an IRB approved single-institution trial.

## Patients and methods

The patient is a 61-year-old woman who presented with an abnormal screening mammogram. It revealed an 8-mm mass in the lower inner quadrant of the left breast. She had a core biopsy performed that showed a Grade 1, infiltrating ductal cancer that was estrogen and progesterone receptor positive.

The patient was seen in consultation by her surgeon and radiation oncologist. Radiation treatment options were explained to the patient, including the standard of care, 6–7 weeks of whole breast radiation therapy, and one week of outpatient APBI. An IRB approved protocol utilizing XB to deliver IORT was also discussed. The patient opted to enroll in the IORT protocol.

On September 3^rd^, 2008, the patient underwent a lumpectomy and sentinel lymph node biopsy. The tumor mass and margins of excision were sent for permanent section, and the identified sentinel lymph node was sent for frozen section. The pathology department reviewed the lymph node specimen at the time of surgery and informed the treating physicians that it was uninvolved by cancer.

**Figure 1:**
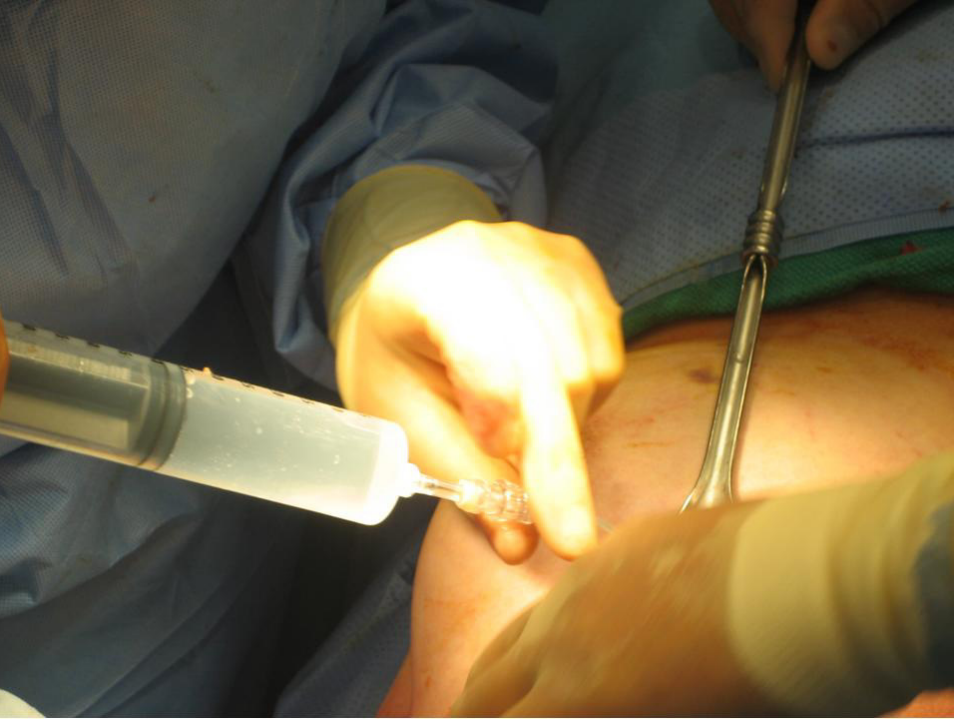
**A CED is inserted through a small incision in the lateral breast and inflated with exactly 40 cc, 50 cc, 60 cc, or 70 cc**.

At that point, additional breast tissue was removed posterior to the lumpectomy cavity down to the depth of the superficial pectoralis fascia to accommodate the chest wall shield. A pliable piece of lead was then temporarily placed over the chest wall to shield the ribs, lung, and heart, from scatter radiation. A cavity evaluation device (CED) was then inserted into the lumpectomy cavity through a small incision in the lateral breast and inflated with exactly 40 cc of saline (Figure 1). The conformity of the CED to the surrounding breast tissue was then evaluated under direct visualization. Conformance was considered to be acceptable if less than 10% of the tissue immediately surrounding the breast tissue was composed of fluid or air and the balloon was tightly opposed to the lumpectomy cavity. Conformance was found to be inadequate, so an additional 10 cc of saline was inserted into the balloon. It was found that conformance was still less than ideal, so an additional 10 cc, or at total of 60 cc, was inserted into the balloon.

Since a CED inflated to 60 cc was found to be the proper size, a 4 × 5 cm XB balloon catheter kit was then opened by the OR staff. The CED was then deflated and removed and a XB was then inserted in place of the CED. If a 40 cc balloon were to be used, a 3 × 4 cm catheter kit would have been opened. The physicist then began calibration of the XB controller. Radiation plans for a 40 cc, 50 cc, 60 cc, and 70 cc balloon were previously developed by CT scanning the XB catheter inflated with saline to the 4 sizes in a water phantom. These plans were then loaded onto USB drives. At the completion of calibration, which typically takes approximately 15 minutes, the physicist then loaded the corresponding radiation plan onto the controller unit.

**Figure 2:**
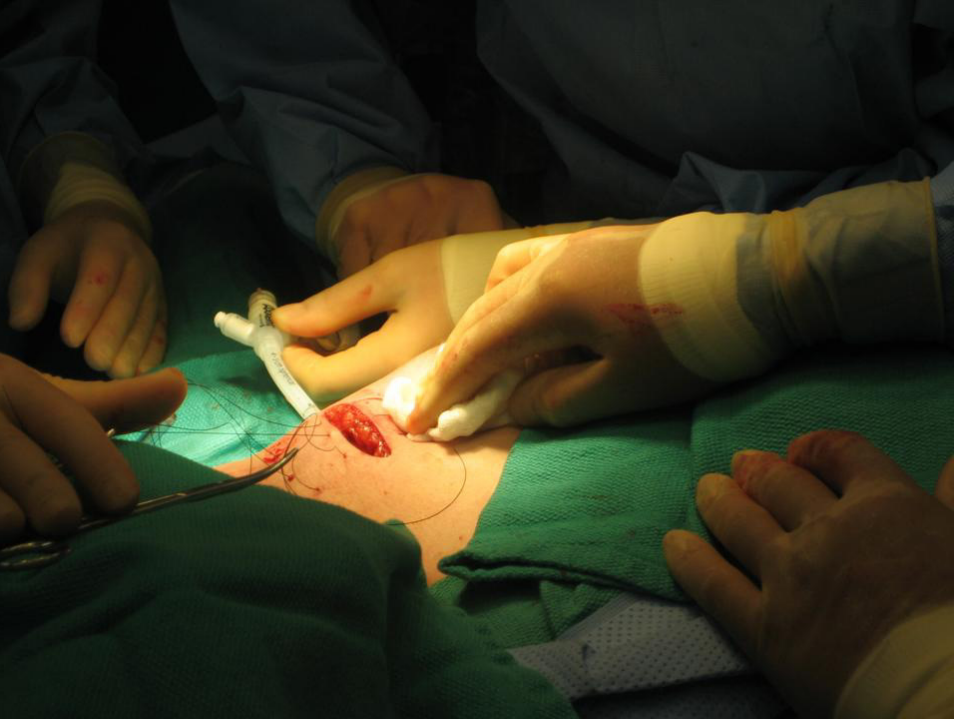
**Retention sutures are placed to potentially increase the balloon-to-skin distance and to more closely approximate the balloon to the surrounding breast tissue**.

During machine calibration, retention sutures were placed in the breast tissue superficial to the inflated balloon (Figure 2). The retention sutures serve to build-up the subcutaneous tissue to potentially increase the balloon-to-skin distance and to more closely approximate the balloon to the surrounding breast tissue. An intraoperative ultrasound was then performed to evaluate the balloon-to-skin distance and the degree of air and/or fluid in the breast tissue surrounding the balloon. For the purposes of this protocol, a minimum of 1-cm balloon to skin distance is required. If the patients are found to have ≥ 0.7-cm and < 1.0-cm of balloon-skin-distance they are offered outpatient balloon-based ABPI. If patients have a balloon-skin-distance of < 0.7-cm, balloon-based brachytherapy is aborted. If patients are found to have large air pockets surrounding the balloon on ultrasound, the balloon is inflated by 10 cc to the next largest size and re-evaluated via ultrasound.

**Figure 3:**
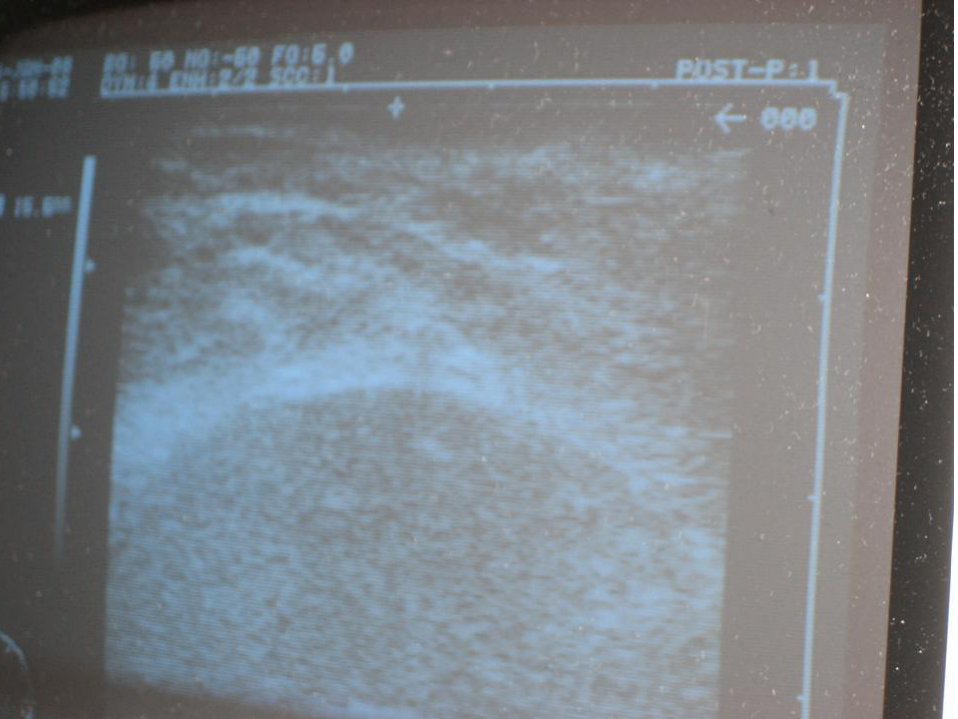
**Ultrasound image showing a balloon-skin-distance of 1.6-cm and no evidence of air pockets surrounding the balloon**.

**Figure 4:**
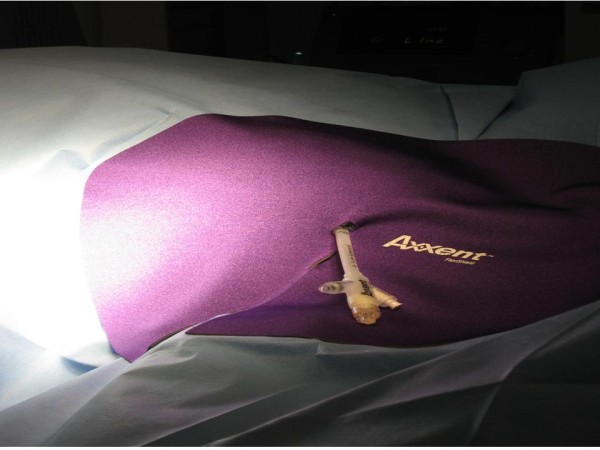
**A sterile drape is placed over the operative field and a FlexiShield™ is placed on top of the drape to minimize radiation transmission**.

The patient was found to have 1.6-cm balloon-to-skin distance and no air pockets surrounding the balloon, so the decision was made to proceed with IORT (Figure 3). A second sterile drape was then placed over the operative field, so that the radiation therapy could be delivered without contaminating the field. The catheter end was passed through a hole in the drape and a FlexiShield™ (FS) was placed on top of the drape (Figure 4). The FS is a lead-equivalent material, which serves to decrease transmission to the patient and hospital staff.

## Results

**Figure 5:**
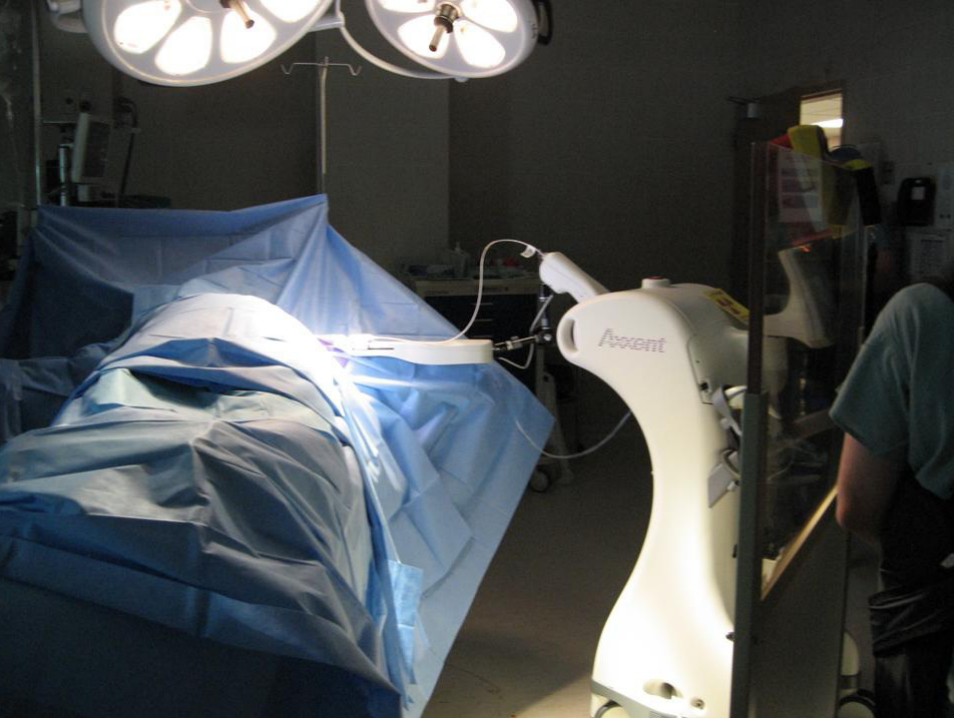
**A radiation dose of 20 Gray to the balloon surface is delivered in approximately 20 minutes**.

The radiation oncologist then broke scrub, attached the XB controller to the catheter, and inserted the radiation source into the balloon. The radiation therapy was then initiated. A total of 20 Gy to the balloon surface was delivered in approximately 20 minutes (Figure 5). The anesthesiologist, surgeon, and radiation oncologist remained in the room during the radiation delivery either wearing lead aprons or standing behind a mobile radiation shield. At the completion of the radiation, the balloon and temporary chest wall shield were removed and the incisions were closed. The duration of the entire procedure including lumpectomy, sentinel lymph node biopsy, balloon catheter placement, radiation therapy, and closing the incisions was approximately 2 hours.

Margin status was assessed by permanent section after the completion of the surgery. Negative microscopic margins are required in the protocol for treatment with IORT alone. Patients who are found to have microscopically positive margins of excision are offered re-excision and whole breast EBRT. The patient was found to have margins of excision of over 2-mm

## Discussion

XB is a novel form of balloon-based APBI, which uses an electronic source to generate kV x-rays. This form of APBI eliminates the need for an HDR afterloader device, heavy shielding, and regulations associated with radiosotope handling and disposal. In addition, XB increases the number of settings in which radiation can be performed. Early clinical reports have shown this technique to be feasible and associated with minimal acute toxicity [12]. Also, XB has been compared to MS (a more established form of balloon-based APBI) and found to offer equivalent target volume coverage with increased normal tissue sparing [16]. To date and since FDA clearance, XB has only been used for outpatient treatment of breast and endometrial cancer. The mobile nature of the XB controller, as well as, the limited shielding requirements make XB a logical modality to be utilized for IORT in the treatment of early-stage breast cancer.

Delivering IORT treatment in a single session is not a new concept. Older IORT techniques involved cumbersome machinery or required custom-built heavily shielded operating suites. Other institutions have performed IORT by transferring patients from the operating room to the radiation oncology department during surgery.

Newer devices for the delivery of IORT utilize smaller, more mobile technology. Two modern mobile linear accelerators have been developed which use 4–6 MeV electrons to deliver a physical dose of approximately 21 Gy [15,17]. An additional device used to deliver IORT is the Intrabeam™ (IB) device, which like XB, uses a 50 kV x-ray source. The IB device delivers 20 Gy at the surface of a solid spherical applicator and approximately 5 Gy at 1-cm depth [18,19]. This is in contrast to XB IORT, which delivers 20 Gy at the surface of the balloon applicator and 9–10 Gy at 1-cm depth. It is unknown whether the increased dose at 1-cm depth associated with XB will have a clinical benefit or possibly increase the risk of toxicity. XB and IB methods of IORT are compared in Table 1.

**Table 1:** A comparison of IB and XB methods of IORT

	**Source**	**Dose at Surface**	**Dose at 1-cm Depth**	**Applicator Type**	**Treatment Time**
**IB**	50 kV x-rays	20 Gy	5 Gy	Solid Spherical	20 – 45 minutes*

**XB**	50 kV x-rays	20 Gy	9–10 Gy	Balloon Catheter	17–26 minutes*

IB = Intrabeam™, XB = Xoft™, kV = kilovoltage, Gy = Gray.

* – Treatment time is dependant on applicator diameter used

Long-term data regarding the safety and efficacy of IORT are not available. The TARGIT trial is a phase III prospective, randomized trial comparing single fraction IORT delivered with IB to conventional whole breast EBRT. Sixteen international institutions are enrolling patients in the trial. Eligible patients include patients ≥ 35 years of age with T1-T3, N0 tumors eligible for BCT. Patients with multi-focal or multi-centric lesions, clinically positive lymph nodes, extensive intraductal component, or invasive lobular cancers are not eligible for enrollment [19].

The results of the TARGIT trial will help determine whether IORT is an equivalent alternative to standard whole breast EBRT. If IORT methods, including XB, are established as a standard treatment option, this may allow increased access to BCT, as well as, improved quality of life and decreased medical costs for patients with a diagnosis of early-stage breast cancer.

## Conclusion

IORT utilizing XB is feasible and can be accomplished in a total procedure time of approximately 2 hours. To date, there are no long-term results utilizing this technique, and patients continue to be enrolled as part of an IRB approved single institution trial. Further research on XB and other methods of IORT is needed to establish clinical efficacy and safety for patients with early-stage breast cancer.

## Abbreviations

BCT: Breast conserving therapy; EBRT: External beam radiation; APBI: Accelerated partial breast irradiation; MS: MammoSite; Ir-192: Iridium-192; XB: Xoft Axxent Electronic Brachytherapy; kV: kilovoltage; ASCO: American Society of Clinical Oncology; IORT: Intraoperative Radiation Therapy; CED: Cavity Evaluation Device; FS: FlexiShield™

## Consent

Written consent for publication was obtained from the patient.

## Competing interests

Dr. Dickler is on the Scientific Advisory Board for Xoft, Inc.

Dr. Francescatti is the Surgical Medical Director for Xoft, Inc.

## Authors' contributions

AD was the treating radiation oncologist and developed the radiation portion of the technique. IO was the primary surgeon and helped to develop the surgical technique. DF was the assisting surgeon and helped to develop the surgical technique. All authors read and approved the final manuscript.
